# Better understanding the ethical and social issues associated with social media uses in physiotherapy: A critical interpretive synthesis

**DOI:** 10.1177/20552076261487056

**Published:** 2026-09-07

**Authors:** Pauline Lemersre, Jonathan Gervais-Hupé, Alexandre Mathieu-Fritz, Annie Carrier, Arthur Filleul, Nathan Bourges, Anne Hudon, Diana Zidarov

**Affiliations:** 1 School of Rehabilitation, Faculty of Medicine, 5622 Université de Montréal (UdeM), Montreal, QC, Canada; 2 Institut Universitaire sur La Réadaptation en Déficience Physique de Montréal (IURDPM), 459878 Centre for Interdisciplinary Research in Rehabilitation of Greater Montreal (CRIR) – CIUSSS Centre-Sud-de-Montréal, Montreal, QC, Canada; 3 Centre for Research on Ethics of UdeM, Montreal, QC, Canada; 4 University Gustave Eiffel, CNRS, Ecole des Ponts, LATTS, 27031 Marne-la-Vallée, île-de-France, France; 5 7321 School of Rehabilitation of Université de Sherbrooke, Sherbrooke, QC, Canada; 6 Research Centre on Aging (CdRV), Sherbrooke, QC, Canada; 7 Research Unit in Pragmatic Health Ethics, Montreal Clinical Research Institute, Canada; 8 Bioethics Program, School of Public Health, 5622 Université de Montréal, Montreal, Canada; 9 SCM KAPAB, Lamballe-Armor, Bretagne, France

**Keywords:** physiotherapy, social media, ethical issues, critical interpretive synthesis

## Abstract

**Introduction:**

Social media uses by physiotherapists are proliferating (e.g. interprofessional collaboration, publicity, information dissemination). While offering opportunities for health democratization, these practices raise ethical and social issues such as cyberincivility and digital literacy-related inequalities, necessitating deeper ethical and social reflection in physiotherapy. Critical literature reviews are useful to develop an evidence-informed theoretical framework to inform ethical reflections.

**Methods:**

This review employed critical interpretive synthesis to examine ethical and social issues in physiotherapists’ social media uses, searching Medline, Embase, CINAHL, and Web of Science (2000–2025), yielding relevant studies via PRISMA-guided screening. Inductive data extraction and analysis were used to generate synthetic constructs through iterative critique, integrating evidence from empirical and non-empirical studies on this topic.

**Results:**

Out of 4547 studies, 28 included studies reveal eight key ethical and social issues in physiotherapists social media uses: privacy concerns and confidentiality, conflict of interest and transparency, cyberincivility and professional misconduct, risks of blurred boundaries between professional and personal spheres, power imbalance during digital interactions with vulnerable populations, risks of uncontrollable and overloaded digital information dissemination, disinformation and misinformation in professional online communication, and risks of jeopardizing intellectual property rights. The resulting conceptual framework prompts reflection on developing digital professionalism in physiotherapy centred on cybercivility and accountability. Fostering digital professionalism requires training professionals and students with digital literacy as a core competency and developing and disseminating clear guidelines.

**Conclusion:**

This review proposes deeper ethical reflections on social media uses in physiotherapy. It emphasizes the need for empirical research to address gaps, enhance reflexivity, and advance digital professionalism.

## 1. Introduction

Social media or the Web 2.0, a concept developed in the early 2000s, is characterized by techniques or functionalities facilitating user interaction and collaborative data creation on the internet.^1^ Examples of social media tools include social networking platforms, wikis, podcasts, blogs, and forums. Social media uses have extended into the healthcare sector.^2^ The concept of Medicine 2.0, introduced in 2009, represents a form of participative healthcare that encourages information and experience sharing between healthcare providers and online users.^3^ Medicine 2.0 practices include diverse initiatives, such as promoting interprofessional collaboration through online communities, facilitating information dissemination via health blogs and medical wikis, and supporting online learning through the sharing of medical videos.^3^ In this context, physiotherapists have increasingly embraced social media. For instance, a 2013 survey conducted in Québec, Canada, revealed that 84% of physiotherapists used Facebook® for both professional and personal purposes.^4^ Physiotherapists engage in various social media activities, including creating wikis, podcasting, social media posts, and blogging.^5^ These practices show a shift in how professional roles are enacted and how health knowledge is shared in the digital age.^3^

The use of social media in physiotherapy responds to the evolving population health needs^6^ and intersects with broader contemporary societal issues in healthcare. Social media offer unprecedented opportunities for health democratization, health promotion, and the development of collaborative care models.^7^ These platforms foster innovative communication channels between physiotherapists, patients, and the wider public, and may help reach new or underserved populations. For instance, individuals of non-white ethnicity are less likely to utilize physical therapy services.^8^ However, a U.S.-based survey found that African American and Latino populations are among the most active social media users and are also most likely than other ethnic groups to use smartphones to access health information.^9^

The integration of social media into healthcare practice is transforming how care is delivered and received^7^ raising significant social issues. While social media can improve access to healthcare resources and information,^2,10^ its use by healthcare providers may exacerbate existing health disparities. Factors such as age, lower educational attainment, particularly in terms of digital health literacy, and socioeconomic status can hinder equitable access to these digital tools.^11^ For example, in Canada, individuals over the age of 55 remain underrepresented among social media users.^11^ Additionally, the generational gap has increased, with Generation Z (generations from 1997 to 2012) favoring platforms like Instagram, Snapchat, TikTok, which are far less used by baby boomers (generation from 1946 to 1964).^12^ This divide leads to unequal access to potentially relevant health resources across age groups. Inequities related to digital literacy may also be further exacerbated, as advanced critical thinking skills and digital competencies are needed for users to distinguish reliable information from misinformation. Indeed, studies indicate that up to one-third of health-related content circulating on social media has been found to be inaccurate or misleading^13^ and concerns persist about its quality and reliability.^14–17^

Ethical issues also relate to the heightened risk of undermining patients’ confidentiality,^18^ which poses serious threats to privacy and data protection.^15,16,19^ Healthcare providers may also struggle to maintain clear boundaries between their personal and professional identities online, leading to blurred professional limits.^4,15,20,21^ Cyberincivility and unprofessional online conduct represent other major ethical issues in digital spaces.^2,22–26^ Examples include the use of offensive language or discriminatory remarks,^2,18^ and the posting of sexually explicit images or depictions of substance use (alcohol, drugs),^16,23–25^ all of which can undermine patient trust and potentially cause harm. As online physiotherapy services evolve, new ethical concepts such as ‘digital professionalism’ have emerged to guide practice.^21^

Despite the widespread integration of social media in physiotherapy, some questions remain unanswered. What is the full range of ethical and social issues related to the use of social media in physiotherapy? How are these issues addressed in scientific literature? How can they be mobilized to inform physiotherapists’ professional practice on social media? In the absence of clear guidance on navigating these practices, a comprehensive examination of their related ethical and social issues is essential.^4^ Adopting a critical perspective is crucial to uncover new avenues for reflection and to inform future practice and research. A critical review of existing literature can help identify knowledge gaps and deepen our understanding of the ethical and social issues surrounding physiotherapists’ uses of social media.^27^

### 1.1. Objectives

The objectives of this review are: 1) to critically appraise the scientific literature on the ethical and social issues related to physiotherapists uses of social media, and 2) to develop an evidence-informed theoretical framework to enhance the understanding and mobilization of these issues in the context of its varied uses in physiotherapists’ professional practice.

## 2. Methods

This review followed the critical interpretive synthesis method (CIS) as described by Dixon-Woods.^27^ CIS was selected for its suitability in synthesizing diverse forms of evidence and generating theoretical insights relevant to complex and underexplored phenomena.^27^ This protocol has been published previously.^28^

### 2.1. Identification of relevant studies

The concept of physiotherapy encompassed all domains of practice, including health promotion, prevention, and intervention within the broader rehabilitation context,^29^ with the intent to include studies involving physiotherapists across diverse practice settings.

Social media and Web 2.0 referred to interactive, web-based tools that facilitate user engagement, collaboration, and co-creation of content, such as wikis, podcasts, social networking platforms, and blogs.^1^ As ethical and social issues often intersect, this review deliberately integrates both dimensions to offer a comprehensive examination of the multifaceted issues physiotherapists encounter in the context of social media uses. Ethical issues were conceptualized as any situation in which the identification, recognition, or respect of relevant values is challenging, potentially leading to harm or consequential choices for the individuals involved.^30,31^ Within physiotherapy, such issues may emerge from evolving practices in education, advertising, treatment modalities, policy, and communication, and include deontological considerations with legal or disciplinary implications, as well as broader questions of professionalism. Social issues were conceptualized as population-level challenges arising from the inherently social nature of care, including disparities in healthcare access, digital health literacy, ageism, and inequities such as limited internet availability or shifting dynamics in online service delivery.^32^

The search strategy was formulated in collaboration with a health sciences librarian, guided by three central concepts: physiotherapy, social media/Web 2.0, and ethical and social issues. Each of the three concepts was defined and operationalized using relevant MeSH terms and keywords. The search strategy was adapted for each of the four targeted databases: Medline, Embase, CINAHL and Web of Science. Initial inclusion and exclusion criteria are outlined in Table 1. Grey literature and articles published in non-indexed journals were excluded from the review.

**Table 1.** Inclusion and exclusion criteria.

Inclusion criteria	Exclusion criteria
• All types of studies (empirical, non-empirical) • In all countries • In English and French • Published between 2000 and 2025 • Published in indexed journal • Addressing ethical and social issues associated with the uses of social media in physiotherapy	• Uses of social media by healthcare providers other than physiotherapists • Uses of social media in research • Uses of social media by patients alone or the general public without the involvement of a physiotherapist • Uses of other digital technologies (artificial intelligence, telerehabilitation or virtual physiotherapy, connected objects, etc.) alone (not combined with social media) • Evaluation of a clinical intervention using social media (satisfaction, clinical effectiveness, quality, etc.) • Uses of social media to train physiotherapists • Conference abstracts

### 2.2. Selection of studies

All references identified through the database search were imported into the Covidence platform for systematic reviews. Initial screening of titles and abstracts was conducted independently by two reviewers (LP and GHJ), with discrepancies resolved through consensus without the intervention of a third party. Selected articles underwent full-text review, also performed independently, with conflicts addressed collaboratively. The study selection process is illustrated in a PRISMA-compliant flow diagram. Consistent with the critical interpretive synthesis approach,^27^ methodological quality was not formally assessed, instead, studies were included based on their conceptual relevance to the research objective.

### 2.3. Data extraction and analysis

A structured data extraction form was developed in Excel and applied to all included studies. For quantitative data, extracted variables encompassed study characteristics such as author names, countries of the first author, publication year, and study type. For qualitative data, extraction focused on content pertaining to ethical and social issues specific to physiotherapy (whether empirical or conceptual), professional viewpoints, social risks, and broader considerations or recommendations related to social media uses in physiotherapy. In the case of empirical studies, additional details were gathered regarding study design, methodology, and population attributes (including countries of practice and practice settings). Qualitative data were extracted through a longitudinal and inductive analysis process informed by meta-ethnographic techniques, from which themes were systematically derived from each article.^27^

The extraction form was piloted and tested independently by both reviewers using the first two articles to assess the feasibility and consistency. Subsequent data extraction was conducted by the primary reviewer (LP), while the second reviewer independently verified three additional articles for quality assurance purposes. The form was refined iteratively to ensure alignment with the study’s objectives. To support this process, frequent meetings with the research team during data extraction and analysis were done.

### 2.4. Critical data analysis and synthesis

The qualitative data extraction aimed to identify the ethical and social issues associated with social media uses by physiotherapists. They have been critically examined and synthesized by the lead reviewer (LP) using the Critical Interpretive Synthesis approach as outlined by Dixon-Woods.^27^ First, a critical reflection column was added to the data extraction Excel sheet. This section enabled a structured critique of how each included study addressed the identified themes, considering aspects such as content depth, comprehensiveness, conceptual complexity or exhaustiveness. Then, the Excel sheet was imported into NVivo 14 to facilitate further qualitative analysis. Constructs were developed throughout an interpretive synthesis, aiming to generate novel, integrative and critically informed concepts to deepen the understanding of the ethical and social issues surrounding physiotherapists uses of social media. These constructs reflect high-order conceptual insights that transcend individual themes identified across the included articles.^27^ Finally, conceptual links between synthetic constructs were established through an interpretive synthesis argument, yielding a cohesive, theory-informed, and critically grounded understanding of the social and ethical issues associated with social media uses in physiotherapy. Given their limited conceptualization in included articles and to enhance clarity, ethical and social issues have been synthesized together without distinction. Nevertheless, we consider a comprehensive social perspective entails initially analyzing an issue by identifying its structural, historical, or organizational causes, while consistently situating individuals within their group affiliations and positionalities (e.g., class, age, gender, digital status) and subsequently examining how these dimensions concretely shape lived situations. Complementarily, an ethical perspective involves analyzing the same issue through clashing values at various scales (micro, meso, macro), such as justice, respect, autonomy, non-discrimination, or transparency, and investigating how actors articulate these tensions, discuss them intersubjectively, and position themselves in their decision-making and practices.

## 3. Results

**Figure 1.**
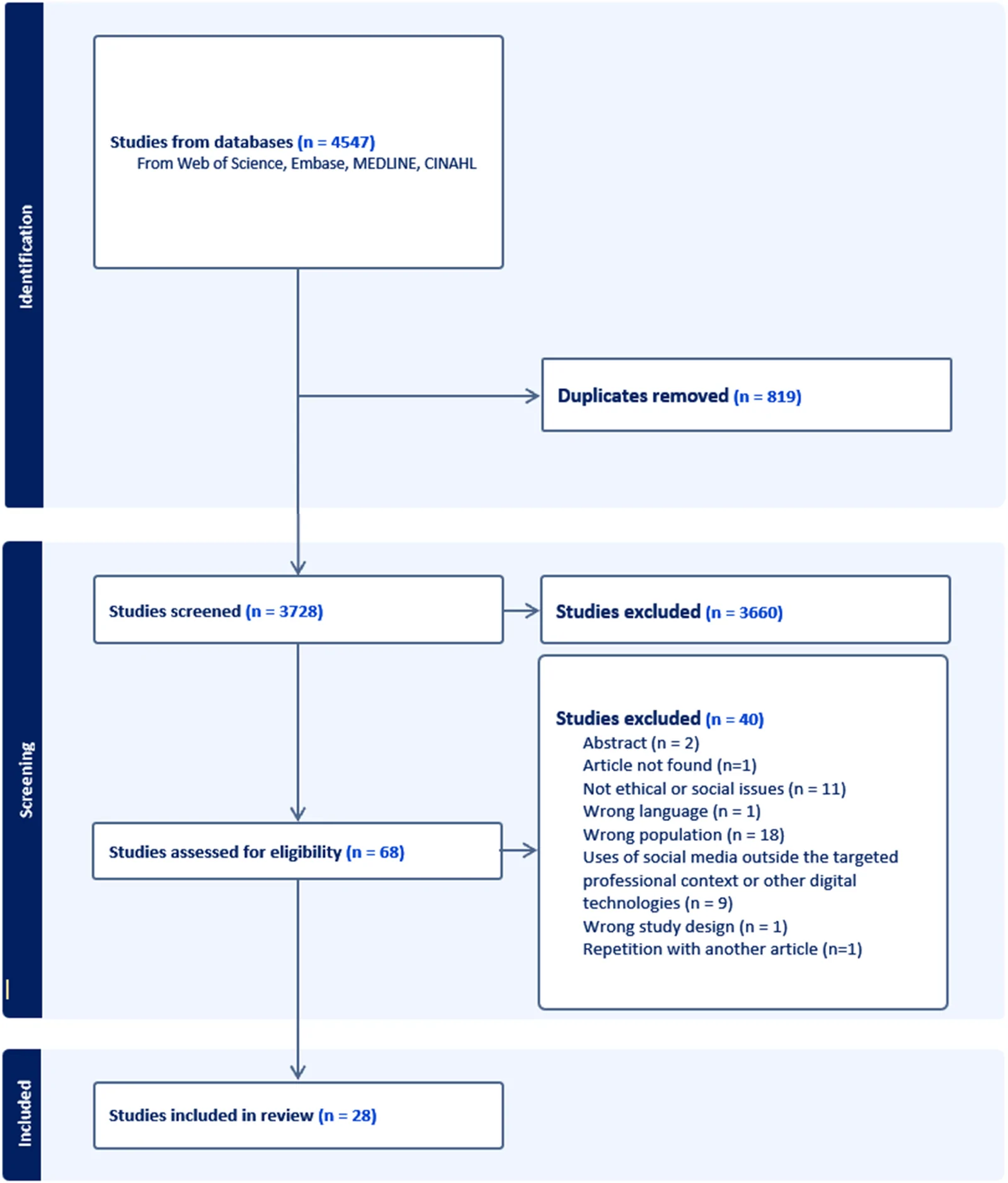
Study selection flow chart.

For this critical interpretive synthesis, a first literature search was conducted on February 21, 2023, in the databases mentioned above and an update was made on July 14, 2025. A total of 4547 studies were identified, and 3728 were imported into Covidence after duplicate removal. Of these, 28 studies met the eligibility criteria and were included in the final sample for analysis. Figure 1 shows the detailed study selection flow chart.

The reading corpus consists of 10 empirical studies, 1 secondary study and 17 opinion pieces, position papers, ethical analysis, or reflective essays. The first authors of the articles originate from the United States (n = 12), the United Kingdom (n = 4), Canada (n = 3), New Zealand (n = 3), India (n = 1), Italy (n = 1), Morocco (n = 1), Sweden (n=1), Pakistan (n=1) and Brazil (n=1). Ethical and social discussions on social media uses in these articles predominantly reflect Western perspectives. While most publications focus on physiotherapy providers (n = 22), some also address physiotherapy students (n = 6). The main topics of articles include recommendations for appropriate social media uses (n = 7), advocacy and promotion of social media opportunities in physiotherapy (n = 6), exploration of current social media practices (n=9), the development of ethical reflections on the topic (n = 5) and the development of an instrument to assess digital professionalism (n=1). Articles included in this review are presented in Appendix 1.

**Figure 2.**
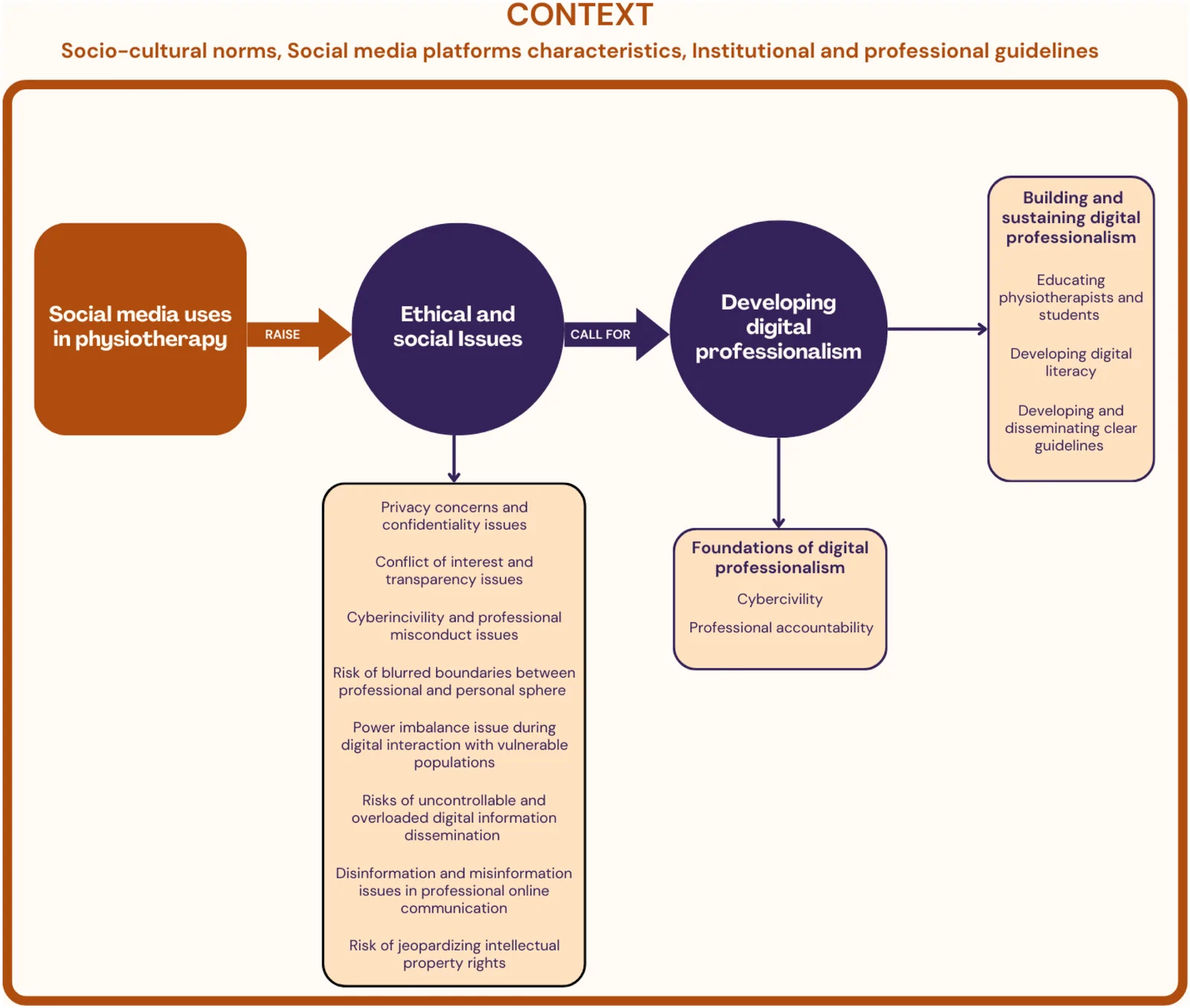
Framework of ethical and social issues associated to social media uses in physiotherapy.

Eight key ethical and social issues are discussed in the literature. Several contextual factors shape the emergence of these issues and were discussed within the included articles. Some of them highlighted the disinhibiting nature of social media, which may prompt physiotherapists to engage in behaviors they would otherwise consider inappropriate in professional settings,^25,33^ creating a potential misalignment between personal values and online conduct.^25^ Platform features such as algorithmic filtering, information distortion, and abbreviated communication formats further influence user behavior and contribute to ethical and social challenges.^34,35^ Newly qualified professionals appear particularly vulnerable, as they are more active on social media than their more experienced colleagues.^23^ These risks are exacerbated by the limited evidence base guiding online professional conduct and the absence of clear institutional and professional guidelines, resulting in an ambiguous landscape where norms surrounding appropriate online behavior remain poorly defined.^4,21,33,35–42^ Our analysis reveals reflection on developing digital professionalism in physiotherapy centred on cybercivility and accountability as foundations. Building and sustaining digital professionalism requires training professionals and students with digital literacy as a core competency and developing and disseminating clear guidelines. Figure 2 presents a framework synthesizing our article analyses and helping to better understand the ethical and social issues associated with social media uses in physiotherapy. This framework suggests that social media uses in physiotherapy raises a set of eight interrelated key ethical and social issues. These concerns call for the development of digital professionalism. Digital professionalism refers to the standards, behaviours, and responsibilities that guide physiotherapists both in person and online. Cybercivility and professional accountability form the foundation of digital professionalism. At the professional level, building and sustaining digital professionalism requires the development of education for physiotherapists and students, the development of digital literacy, and the development and the dissemination of clear professional guidelines. These uses, issues, and the digital professionalism are shaped by contextual factors including socio-cultural norms, social media platform characteristics, and existing professional and institutional guidelines. These factors influence how ethical and social issues and digital professionalism are defined, expressed, and understood in practice.

## 4. Issues raised by social media uses in physiotherapy

Eight key ethical and social issues associated with physiotherapists uses of social media were identified. In most articles, ethical and social issues were not explicitly defined, rather, they were mentioned or occasionally illustrated through examples. This section synthesizes these social and ethical issues and highlights the recurrent ethical questions raised across the corpus.

### 4.1. Privacy concerns and confidentiality issues

Breach to privacy and confidentiality are among the most frequently cited ethical issues in the literature regarding social media uses by physiotherapists.^4,21,23,25,33,38,40,43–49^ Both physiotherapists and patients often neglect to appropriately configure privacy settings on professional pages and personal profiles,^4^ with these settings frequently chosen hastily or without careful consideration.^21^ This lack of rigor poses significant risks to the security of shared and stored information.^38^ Several documented practices may result in breaches of confidentiality, such as sharing images or videos of patients that directly violate their privacy.^23,46^ Even seemingly innocuous disclosures, whether medical or personal, can inadvertently contain identifiable information that compromises patient confidentiality like ages, cities, professions, etc.^25,33,38,45^ Furthermore, seeking information about physiotherapy patients or colleagues online, whether current, former, or prospective, also raises concerns.^23,25^ For instance, viewing patient profiles for purposes such as tracking progress, satisfying curiosity, or gathering additional background information may be considered unethical involving respect for privacy and informed consent.^33^

### 4.2. conflict of interest and transparency issues

Several articles highlight conflict of interest and transparency issues.^23,33,37,43,46,49–52^ A conflict of interest (COI) is defined in the included literature as a post on social media including professional contact information for commercial purposes, or a profile promoting products or services, either independently or through partnerships (e.g., within the past two years). Additionally, pages or profiles presenting selective reporting of only positive outcomes from cited sources, excluding negative or non-significant findings, were also regarded as potential COIs due to selection bias.^52^ Some uses of social media by physiotherapists are described as being driven by economic motivations,^37,43,46^ including brand development (e.g., for clinics), attracting new patients, and pursuing a return on investment.^46^ The literature also highlights professional uses such as offering private courses, forming corporate partnerships, or promoting specific rehabilitation approaches.^51^ Such practices may create conflict of interests by challenging the conventional distinction between public domains (e.g., health care system) and private interests (e.g., private training organizations or commercial industries).^33^ Additionally, some social media content primarily aims at marketing or endorsing specific products,^37,43^ raising concerns about transparency and potential COIs.

### 4.3. Cyberincivility and professional misconduct issues

The literature documents numerous instances of cyberincivility by physiotherapists on social media platforms,^37^ with a particular focus on physiotherapy students.^23,25,34,37,53^ Manifestations of cyberincivility include biased and stereotypical remarks,^37^ demeaning comments toward patients,^37^ and discriminatory content such as homophobic, misogynistic, or racist language.^23,54^ Some articles also report inappropriate sexual behavior online,^42^ including sexually suggestive messaging and, in extreme cases, the initiation of sexual relationships with patients.^33^ Studies further describe recurring low-quality posting behaviors by physiotherapists, such as personal attacks, rude or offensive commentaries, the use of profanity, and posts with inappropriate tone or content.^23,25,34,37,41,45,51,55^ Uncivil behaviours may lead to disciplinary sanctions^33^ such as litigation,^33,46^ deregistration from professional boards, or termination of employment, each with the potential to impact the profession’s reputation^4,23,33,39,46^ and erode public trust in physiotherapists.^23,38,43^ At the individual level, a physiotherapist’s personal social media behavior, particularly when it involves unprofessional content, may further undermine their perceived credibility^33,35^ and raise doubts about their competence or suitability to provide care.^55^ This erosion of trust can also have serious implications for care delivery (e.g., damaging the therapeutic relationship) and may potentially impact patient adherence to treatment recommendations.^23^ In a virtual context, typical of online environments, messages can easily be misinterpreted.^23,25,33^ Moreover, perceptions of acceptable social media behavior may vary across audiences,^25^ making it difficult to establish clear professional standards.

### 4.4. Risk of blurred boundaries between the professional and personal sphere

Online identity constitutes a significant challenge, often blurring the boundaries between personal and professional spheres.^4^ This can harm professional integrity and the protection of privacy. Separating professional and personal information or using different accounts does not guarantee a clear distinction between a user’s personal and professional image. Content shared on personal accounts can still be viewed by patients or employers and can have consequences (e.g., disciplinary sanctions). Blurred boundaries between personal and professional relationships are a recurring concern, particularly in instances where physiotherapists develop online friendships with patients.^4,21,33,35,36,39,41,42,45,48,52^

### 4.5. Power imbalance issues during digital interaction with vulnerable populations

Some studies raise social questions about online interactions between physiotherapists and minors, especially concerning age restrictions and consent to use social media platforms.^21,40^ Given their increased vulnerability, children are particularly susceptible to online influence, whether positive or negative, with some users exerting considerable impact on younger audiences.^51^ Furthermore, hierarchical relationships, such as personal connections between physiotherapists and their patients or employers, may create implicit pressure to engage in social or digital interactions.^4^

### 4.6. Risks of uncontrolled and overloaded digital information dissemination

Controlling the dissemination of information shared on social media platforms presents significant challenges.^4,33,35,38,40,44,45,51,54^ Once content is published, it often becomes permanent, particularly through features such as screenshots that allow indefinite preservation and redistribution.^33,45^ Even in private or closed groups, it is virtually impossible to prevent members from copying or sharing content beyond its intended audience.^4^ Once information is disclosed, regulating how it is interpreted or used by others becomes exceedingly difficult.^40,44,51^ Additionally, the vast volume of content generated on social media platforms can contribute to information overload,^35,41^ to the circulation of contradictory viewpoints^51^ and spread of redundant, uncontrolled, and often self-referential information. These problems can lead some providers to reduce their engagement in online professional communities^51^ or make it difficult for both physiotherapists and patients to disengage from social media platforms (e.g., switch off social media platforms or take breaks).^35,38^

### 4.7. Disinformation and misinformation issues in professional online communication

Disinformation and misinformation represent recurring challenges highlighted throughout the literature.^34,39,43^ Discussions in clinical online posts often show minimal interaction (e.g., likes or emoji reactions), limited reciprocal engagement (e.g., reasoning or discussion), with content frequently being self-referential^45,51^ and can lead to dissemination of incorrect information. The scope of knowledge exchanged through social media tends to be narrow and may reflect biased perspectives.^42,51,52^ Furthermore, social media platforms significantly contribute to the dissemination of false information, including fake news, fabricated scientific claims, and pseudoscientific content.^34,39^

### 4.8. Risk of jeopardizing intellectual property rights

Intellectual property rights were mentioned in only one article as an issue related to the use of social media in physiotherapy, but this concept was not defined, described, explored in depth or illustrated with examples.^21^

## 5. Developing digital professionalism

Several articles included in this review highlight the need for guidance and propose numerous suggestions, guidelines, and recommendations, highlighting the concept of digital professionalism as a key framework. Digital professionalism involves upholding common core values, such as responsibility, respect, and privacy that also characterize offline professionalism, while applying them to the unique challenges and opportunities of digital tools and social media. It highlights opportunities for their effective and positive use while also recognizing policy and ethical considerations related to their professional uses.^21^ Digital professionalism in physiotherapy practice is influenced by both individual circumstances^39^ and sociocultural norms.^23,25,39^

### 5.1. Foundations of digital professionalism

#### 5.1.1. Cybercivility

Cybercivility is defined as fostering a positive, respectful, and inclusive digital culture that upholds mutual respect, sensitivity, and professional demeanor in all online exchanges. It is primarily concerned with interpersonal aspects and maintaining civility and cooperation in electronic communications^37^ and can be linked with upholding ethical conduct online.

Despite the emergence of new challenges associated with social media uses in physiotherapy, some authors argue that these platforms do not fundamentally alter existing expectations of professional behavior.^21,46^ Physiotherapists are already trained to uphold principles such as confidentiality and professionalism in their clinical practice,^35^ and these standards are equally applicable to online settings. Moreover, issues like transparency for example are not new and predate the digital era, suggesting that social media simply introduces new contexts in which enduring professional obligations should be maintained.^35^ According to these authors, existing expectations regarding professional behaviours and current ethical code should be useful and applied in online social media uses.

A growing body of literature from our sample emphasizes that the use of social media in physiotherapy requires respectful, responsible, and ethical conduct in all forms of online communication or interaction.^4,21,33,36,38,39,45,46,48,50^ First and foremost, authors mentioned that physiotherapists should uphold core principles of medical ethics, autonomy, beneficence, nonmaleficence, and justice, while engaging with social media within adherence to established professional norms and guidelines.^21,33,36,38,39,45,46,48,50^ For instance, physiotherapists are expected to take responsibility for their online content and opinions, and to avoid anonymous participation on social media platforms about physiotherapy subjects.^21,48^ Applying these ethical principles in digital contexts may require a reconceptualization of how these standards are applied in online communication.^21,33^ Furthermore, before engaging in social media activities, physiotherapists should critically examine potential tensions between broader societal health needs and their individual professional objectives, ensuring that their online presence supports public benefits.^35^ To promote equitable access to digital information, some articles argue that physiotherapists should use clear and inclusive language, assess whether their intended audience can engage effectively with social media content, and prioritize open-access pages or groups where information is freely available.^33,36,38,46^ Social media uses should actively protect the well-being of individuals engaging with these platforms by minimizing harm, such as exposure to harmful content or negative interactions.^34,35,38,46,50^ Particular attention should be given to protecting patients. This could be done by carefully considering the multimedia stimuli presented on screens when working with vulnerable individuals, such as patients with traumatic brain injury or cognitive impairments.^38^ If concerns arise regarding a patient’s condition during online communication, physiotherapists should refer to the individual to their primary.^38^ Practitioners should also remain vigilant about the safety of children in online environments.^35^ In addition, clinicians should take steps to protect their own well-being and that of their colleagues.^46^ This involves alerting peers if they share content that appears inaccurate or breaches confidentiality, and ensuring that professional websites or profiles include clear disclaimers.^33,46,50^ Some authors further recommend that, in cases of serious or repeated violations of ethical or professional standards, such behavior should be reported to the appropriate regulatory authorities.^33,46^

#### 5.1.2. Professional accountability

Professional accountability refers to the extent to which healthcare providers are held responsible for patients, colleagues, employers, and society for their behavior, clinical judgment, and decision-making.^48^ For example, physiotherapists have a responsibility to protect both their own privacy and that of their patients when using social media.^4,33,35,39,46,56,57^ This includes the proper configuration of privacy settings and the understanding that all online content should be considered publicly accessible, regardless of platform restrictions.^4,21,33,35–38,46^ Some included articles propose educating both physiotherapists and patients on how to adjust privacy settings to better control the visibility of their personal information.^4,21,33,35,37,46^ While several authors recommend maintaining separate personal and professional profiles,^4,21,33^ this approach does not eliminate the risk of real-world consequences, such as disciplinary sanctions or reputational damage, resulting from unprofessional or inappropriate online behavior even on their “personal” profile.^33,46,49^ In addition, an individual physiotherapist’s online presence can be perceived as representative of the entire profession.^21,33,38^ This collective representation places a responsibility on physiotherapists to uphold professional standards and protect the reputation of the field when engaging in social media platforms.^21,23,33,45^ To meet these expectations, some authors propose that practitioners develop a well-defined professional online identity and manage their digital presence with care and strategic intent.^21,37,38^

Several authors also argue that, despite potential ethical challenges, physiotherapists should actively engage in online communities and participate in social media platforms, suggesting that such engagement may even constitute a professional responsibility.^4,21,34,39,46,49,51,55^ According to these perspectives, a professional presence on social media can yield more benefits than drawbacks.^34^ It allows physiotherapists to disseminate accurate and evidence-based information, counter misinformation, promote public health initiatives, advocate for best clinical practices, and facilitate professional networking.^4,39,44,55^ Social media can also promote equity, for example, by improving access to resources and information, and enhance accessibility to physiotherapy services.^35^ To strengthen their professional practice,^39^ physiotherapists are encouraged to cultivate and sustain a social media presence, sharing high-quality educational content, such as infographics and short-form videos (e.g., Instagram Reels), and leveraging different platforms to reach broader and more diverse audiences.^21,46^

### 5.2. Building and sustaining digital professionalism

#### 5.2.1. Educating physiotherapists and student education

To adequately address ethical and social issues, and to foster digital professionalism in physiotherapy, targeted education (e.g. social media uses, digital literacy, etc.) for both practicing physiotherapists and students seems to be a possible and relevant solution. Several studies in our sample examine approaches to training physiotherapists in web-based communication and cybercivility, highlighting the design and implementation of effective educational programs applicable across diverse international contexts.^4,21,25,33,36,53^ Proposed educational modalities include continuing education workshops, informal “lunch-and-learn” sessions, and webinars,^21,36^ that may be complemented by supplemental resources such as instructional videos^21^ or self-assessment tools for online behavior.^48^ Peer learning is also encouraged, with recommendations to engage with professionals who exemplify positive online behavior and serve as role models within the profession.^35,46^

#### 5.2.2. Developing digital literacy

To develop appropriate social media practices, physiotherapists should acquire digital literacy.^4,25,53^ This competency involves the strategic creation, sharing, and dissemination of online content, such as developing a professional social media plan or writing blogs,^4,53^ while building an engaged audience and clearly defining the goals of one’s digital presence.^53^ It also entails understanding social media uses,^53^ including the ethical and professional use of mobile devices and applications, as well as respecting intellectual property rights.^44^ Additional skills include staying informed about emerging platforms and measuring the success of one’s page or profile using metrics such as follower count, engagement rates, and referral traffic.^44^ A critical approach to social media content is also an important skill,^46,53^ ensuring that blogging and information sharing are grounded in current evidence and thoughtful interpretation.^46,53^ Finally, physiotherapists should regularly monitor and assess their digital presence to ensure alignment with their intended professional identity.^21,33,46,48^

#### 5.2.3. Developing and disseminating clear guidelines regarding the use of social media in physiotherapy

**Figure 3.**
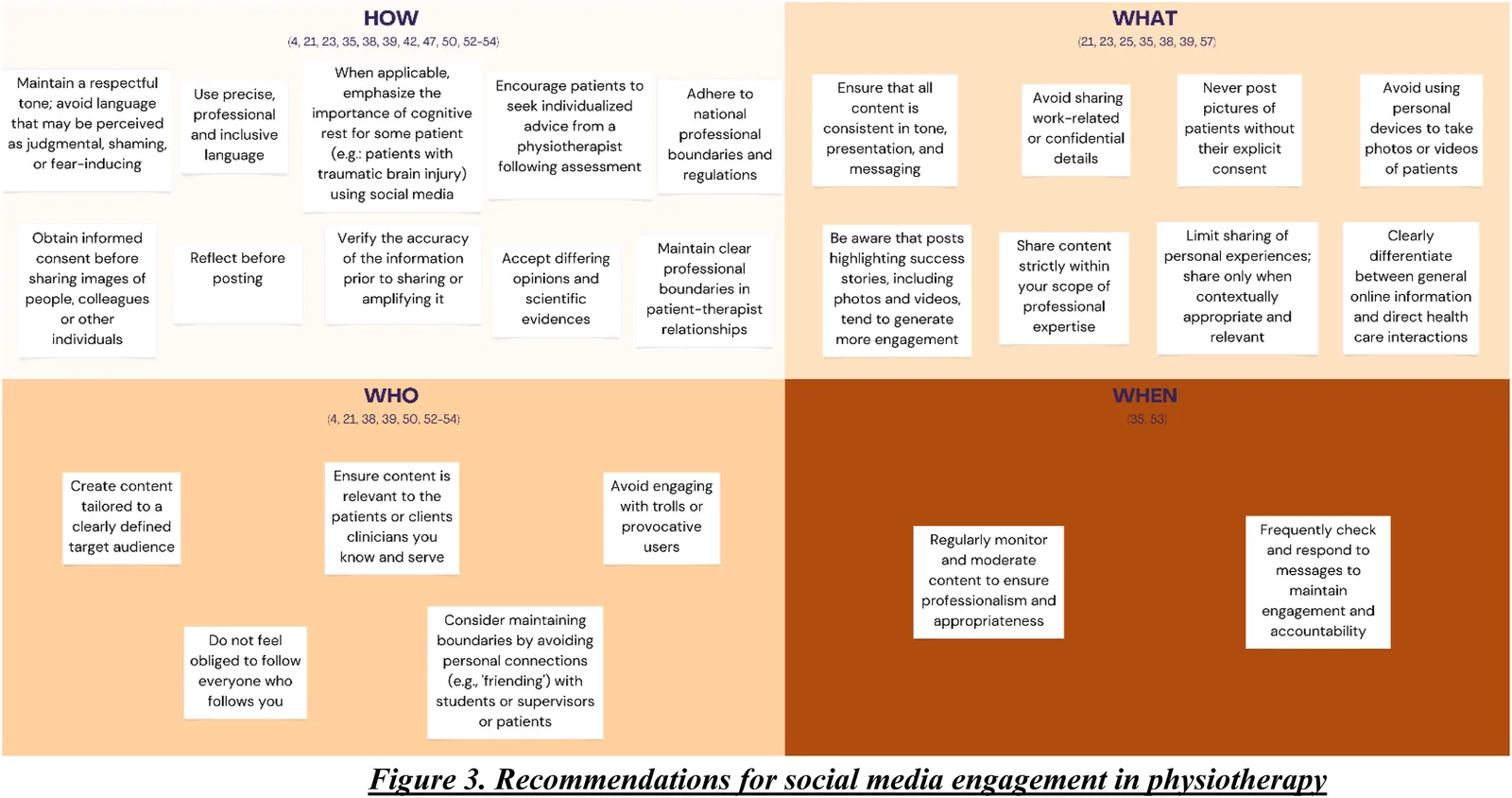
Recommendations for social media engagement in physiotherapy.

Many articles in our sample highlight the need for clear guidelines to support the appropriate use of social media in physiotherapy.^4,21,33,37–42,44^ Such guidelines are useful to ensure that physiotherapists understand their legal and professional obligations when engaging in online activities.^4,21,23,46,49,56^ To support responsible use, healthcare institutions should provide education, guidance, resources, and training opportunities tailored to digital professionalism,^21^ as well as to establish explicit policies governing students’ social media conduct and create safe spaces for discussing complex or controversial issues such as ethical concerns.^23,25,37,42^ Workplaces settings should also establish context-specific social media policies aligned with local ethical, cultural, and legal norms.^33^ Moreover, physiotherapists should remain informed of relevant regulations, licensing requirements, and professional standards, particularly when offering online clinical advice across borders, as cross-border communication does not exempt practitioners from jurisdictional restrictions.^38,50^ Indeed, providing clinical advice or care where one is not licensed may result in legal or professional consequences.^50^ Calls for greater regulatory oversight include the implementation of accreditation processes to oversee physiotherapists use of social media, or the adoption of a self-assessment tools to measure digital professionalism^4,48^ and a range of standards and recommendations for social media engagement in physiotherapy has been proposed across the included studies,^4,21,23,25,33,35,36,38,39,46,50,53,55,58^ and are presented in Figure 3.

## 6. Discussion

The reviewed corpus comprises 28 articles exploring the ethical and social issues related to physiotherapists uses of social media. To our knowledge, this is the first critical interpretative synthesis exploring and critically appraising the literature on this topic.

Our analysis revealed that social media presents a multidimensional and complex landscape for physiotherapists where multiple ethical and social challenges emerge. Maintaining privacy and confidentiality is essential, and physiotherapists should avoid sharing client information online or assuming that privacy settings alone are sufficient protection. The permanence and wide dissemination of digital content contribute to information overload, while the difficulty of retracting or controlling posts creates lasting professional implications from physiotherapists. Transparency issues are amplified when posts promote products, raising potential conflicts of interest, especially when commercial motivations are involved. Instances of cyberincivility and professional misconduct can lead to disciplinary actions and erode public trust in the profession. Disinformation and misinformation spread rapidly on social platforms, complicating knowledge sharing and communication and putting patient safety at risk. Intellectual property and blurred personal-professional boundaries are also persistent risks, requiring careful strategies and self-regulation to limit consequences for physiotherapists and patients. Online interactions may also create power imbalances, especially when engaging vulnerable groups, and necessitate rigorous consent and ethical practices.

These findings are consistent with reflections and findings discussed with regards to social media uses by other health professionals. For example, privacy concerns and confidentiality issues are frequently cited regarding social media uses among other healthcare professionals.^2,15–18,59^ Loss of privacy is also a recognized concern associated with patients’ use of social media for health-related reasons^60^ These issues seem to receive an extensive coverage from healthcare professionals’ writings, bringing to light distinct issues such as ‘patient-targeted Googling,’ the practice of searching for patient information on social media.^15,16,19,59,61^ Although this behavior may stem from a desire to better understand patients, it can also reflect excessive curiosity or even inappropriate voyeurism.^15,16,19,59,61^ Additionally, consideration of peripheral data deriving from online posts, such as location, posting dates, and social interactions, emerges as an important element in discussions of this issue among other healthcare professionals. Followers can infer sensitive information from a professional’s social media profile, thereby heightening confidentiality risks.^62^ Conflict of interests and transparency issues,^16,62–64^ risks of blurred boundaries between professional and personal spheres^2,16–18,59,62,65^ and risks related to the uncontrollable or excessive dissemination of digital information^2,16,19,20,59,62,65^ also frequently arise. Cyberincivility and professional misconduct issues are similarly prominent, with particular emphasis on medical students and nurses.^2,15,16,18,24,64,65^ Disinformation and misinformation in professional online communication also concern other healthcare professionals, underscoring the rapid spread of false information and questioning professionals’ roles and responsibilities in creating online content to counter it.^14–17,59,63^ The quality of information is a major concern for patients when seeking health information on social media. In the United States, one-third of adults perceive a high level of misinformation, and two-thirds report difficulty distinguishing between accurate and inaccurate health information.^66^ Less prominent in our corpus, risks to intellectual property rights are also addressed by other healthcare professionals. Platform policies typically grant social media companies legal rights over posted content, leaving users unable to retain ownership or to adequately protect patient privacy once images are shared.^64^ While our results indicate that online interactions may create power imbalances when physiotherapists engage with vulnerable groups, it is important to note that patients may perceive these interactions as shifting traditional power dynamics between themselves and healthcare professionals. The use of social media can help reduce information asymmetry, foster more balanced communication, and support shared decision-making.^60,67^ Similarly, social media serves as a key lever for caregivers of vulnerable populations (e.g., elderly individuals or children) to challenge traditional power imbalances. It does so by expanding knowledge, enabling new advocacy opportunities (e.g., caregiver influencers), and providing peer support through online groups.^68,69^

The corpus analysis we completed also reveals that described ethical and social issues call for the development of ‘digital professionalism’ in physiotherapy. The fundamental ethical principles applied by physiotherapists in face-to-face settings remain unchanged and should continue to apply online. However, the included articles emphasize how these principles are enacted differently in digital contexts through the concepts of cybercivility and increased professional accountability. To foster digital professionalism, our findings highlight the importance of educating both students and practitioners in physiotherapy. Indeed, our analysis underscores digital literacy as an emergent key competency necessary to ensure ethical, responsible and professional use of social media within the profession.

These findings are consistent with reflections on other health professionals who use social media. Regarding digital professionalism, cybercivility is also a well-developed concept across other healthcare professions, with similar definitions, and is considered important to its development.^2,21,37,48,70^ Professional responsibility on social media is also a central concept discussed in the literature on other health professionals,^2,48,70,71^ reflecting the ethical and legal obligations that persist in digital environments. Education for both practitioners and students are identified as key strategies for fostering responsible digital practices.^2,48,70^ In addition, digital literacy is viewed as an essential competency not only for health professionals but also more broadly for patients.^72,73^ Finally, the need to develop practice recommendations is also highlighted across other health professions^2,70^ with the American Medical Association (AMA) social media guidelines emerging as one of the most frequently cited and commonly used reference documents in this area.^70^

### 6.1. Critical perspectives

Although the articles included in this corpus raise many relevant ethical and social issues with regards to the use of social media in physiotherapy, empirical evidence remains limited. Some articles present a variety of ethical and social issues that are related to the use of social media by physiotherapists, but these issues are often not stemming from empirical research. The issues are rarely defined, remain underdeveloped, and the empirical and reflexive depth of the studies is generally limited. The articles included in this review focus only on the perspectives of physiotherapists and physiotherapy students, excluding other viewpoints such as those of patients, caregivers, or the general public for example. In addition, the corpus predominantly reflects Western perspectives, and the discussion of these issues might differ within a cultural or value system distinct from those contexts. Individualist cultures, such as those commonly observed in Western countries, may shape professional interactions on social media, where privacy concerns are often viewed primarily as an individual Responsibility.^74,75^ In contrast, collectivist cultures, including those commonly observed in Eastern countries such as China or Korea, may place greater emphasis on privacy concerns related to group status, social harmony and collective well-being.^74,75^ In these contexts, professionals may view social media more as a communal workspace that emphasizes information sharing within trusted groups rather than individual distinction (e.g., personal branding).^76–78^ At the same time stronger hierarchical norms^76,77,79^ may shape online professional interactions and influence perceptions of appropriate professional and personal boundaries, including interactions such as friending patients on social media. More empirical researches and critical analysis are required to support the development of adapted, relevant, and inclusive frameworks. Although some recent articles are included, the rapid growth in social media uses for health purposes and the increasing role of artificial intelligence in content creation^79^ have rendered a portion of the literature either outdated (e.g., publications from the 2010s) or on the verge of becoming obsolete (e.g., studies from 2019 or 2020). Indeed, AI is now an integral part of social media platforms, notably through the personalization of content feeds, user data analyzing, automating content creation and publishing, and targeted advertising.^80,81^ These applications are mainly designed to increase user engagement and optimize marketing strategies by enhancing user experience.^80,81^ However, they also intensify privacy concerns, as AI systems rely on the collection and analysis of large amounts of personal data to profile users and shape the content they are shown.^80,81^ In this context, users may not fully understand which data are being collected or how they are being used. AI-driven systems may also amplify discriminatory behaviors already present online by influencing content visibility.^80,81^ In addition, they can facilitate the spread of misinformation and deceptive content, including deepfakes.^80,81^ The growing use of generative AI to generate health-related information on social media is an additional concern.^82^ For example, AI can generate highly credible-looking misinformation that mimics scientific reliability criteria such as false scientific citation.^83^ This issue is especially important because AI-generated content has been shown to generate higher user engagement than human-created content.^82^ Many articles emphasize an urgent need for the development of guidelines, regulatory frameworks, and the development of the concept of digital professionalism in physiotherapy. Their recommendations are often broad generalizations that are rarely based on empirical evidence and may often seem remote, or even detached, from the concrete, local realities encountered by practitioners. Reflections on the application of current codes of ethics to social media uses are generally superficial in our sample, and do not adequately consider the unique characteristics of online platforms, such as their disinhibiting effects and the cross-border nature of online practice (e.g., whether a practice license is required for a given jurisdiction).^84^ Perspectives focused on commercial opportunity and marketing uses in our sample also tend to overlook questions of ethics, deontology, conflicts of interests and equitable access.

Even though our corpus analysis revealed a variety of ethical and social issues consistent with reflections from other health professionals, some key concerns, such as user profiling^63^ and the role of algorithms in distorting online reality (e.g., filter bubbles, echo chambers),^85,86^ are either absent or not yet considered in the context of social media uses in physiotherapy. User profiling, for example, which involves the automated analysis of personal data to identify or categorize individuals, poses notable risks. These include potential impacts on healthcare professionals (e.g., reputational harm, exploitation of data for targeted marketing) as well as risks for the public (e.g., stigmatization, discriminatory practices in employment or insurance contexts).^63^ Moreover, the algorithms underlying social media platforms tend to generate filter bubbles and echo chambers by directing users toward information similar to what they have already engaged with, at times granting increased visibility to unreliable content and exacerbating the polarization of ideas.^61,85,86^

Our findings highlight that the rapid rise of social media has outpaced existing guidelines and discussions on online physiotherapy practices resulting in ambiguous advice and guidance, and inconsistent regulatory oversight, highlighting the pressing need for evidence-informed, cultural and professional-specific standards.

### 6.2. Perspectives for further reflection

A deeper understanding of ethical and social issues raised by social media uses in physiotherapy, particularly how they arise and their social consequences, can inform collective reflection and guide professional actions. It would be important for physiotherapists to develop a stronger ethical sensitivity toward social media practices and to train in digital professionalism to support a more responsible and equitable social media engagement. Recognizing complex social factors such as implicit biases, cultural impositions, or organizational constraints that affect their online uses, could also support this endeavor.^31^ These reflections are important for shaping both education and practice standards. It is imperative to pursue rigorous empirical research on this topic to move beyond the predominant descriptive or prescriptive approaches, to deepen our understanding of currently identified and potentially still unforeseen ethical issues and develop and to elaborate and evaluate concrete strategies to support the ethical use of social media by physiotherapists.

### 6.3. Limitations and strengths of the study

Our study has several limitations. The exclusion of grey literature may have limited the exhaustiveness of the results, potentially omitting relevant ethical and social issues. A substantial proportion of included studies consisted of non-empirical sources, which limits the empirical strength of the evidence base and the robustness of some conclusions. Additionally, the conceptualization of the results relied primarily on the interpretation of a single researcher, which might have limited the depth of the critical synthesis process. However, frequent meetings with the research team during data extraction and analysis helped mitigate this limitation. The critical interpretative synthesis approach is relatively recent, and guidance remains limited. Publishing our protocol^28^ increased transparency regarding the planned methods, demonstrated that the synthesis followed a systematic and thoughtful methodology, and provided a reference for future researchers using this approach.

The conceptual framework developed in this article provides a comprehensive understanding of the ethical and social issues associated with social media uses in physiotherapy to date in the literature, and its associated critiques highlight numerous gaps that still need to be filled. This study on physiotherapists may also offer useful insights for researchers focusing on other health professions. Methodological strengths include the systematic approach, which ensured diversity among the sources analyzed. By integrating studies with a diversity of methodologies and settings, the study offers a more comprehensive view of the topic. Finally, the iterative and reflective process allowed for a critical interpretation, thereby laying a stronger foundation for future research on this topic.

## 7. Conclusion

In conclusion, this first critical interpretative synthesis of ethical and social issues associated with physiotherapists uses of social media highlights the large diversity of these issues. This review identified eight key issues: 1) privacy and confidentiality, 2) conflicts of interest and transparency, 3) cyberincivility and professional misconduct, 4) blurred boundaries between professional and personal spheres, 5) power imbalances in digital interactions with vulnerable populations, 6) risks of uncontrollable and overloaded digital information dissemination, 7) disinformation and misinformation in professional online communication, and 8) risks of jeopardizing intellectual property rights. Drawing on these findings, this review proposes an evidence-informed theoretical framework centred on the development of digital professionalism in physiotherapy, with cybercivility and professional accountability as foundations of digital professionalism. Building and sustaining digital professionalism include the integration of targeted education for practicing physiotherapists and students, the recognition of digital literacy as a core professional competency, and the development and disseminating of clear, evidence-informed guidelines tailored to online, social media practice. Ethical issues are often poorly defined and insufficiently theorized, with the majority of perspectives arising from Western contexts. This conceptual framework prompts reflections on ethical and responsible online practices in physiotherapy. It deepens understanding of the ethical and social issues tied to social media use while highlighting building and sustaining digital professionalism to address them. Findings from this critical review also emphasize the importance of raising awareness among physiotherapy students and professionals regarding digital professionalism. While this review proposes an integrative framework to structure these issues, further researches are needed to test, refine, and validate this framework across diverse practice and cultural contexts.

## Appendix

**Appendix 1.** Table of articles included in the critical interpretative synthesis.

Article (n = 28)	Authors	Study type	Country	Year	Aim of the study	Study design	Main topic
Current Uses (and Potential Misuses) of Facebook: An Online Survey in Physiotherapy^4^	Maude Laliberté; Camille Beaulieu-Poulin; Alexandre Campeau Larrivée; Maude Charbonneau; Émilie Samson; Debbie Ehrmann Feldman	Quantitative study	Canada	2016	To explore professional and personal use of Facebook by physiotherapy professionals in Quebec	Cross-sectional exploratory study	Exploration of current social media practices
Professionalism in a Digital Age: Opportunities and Considerations for Using Social Media in Health Care^21^	Kendra Gagnon, Carla Sabus	Perspective article	USA	2015	To provide a broad overview of the emergence of social media in society and healthcare, to explore the policy implications of organizational adoption of social media in healthcare, and to propose individual opportunities and guidelines for social media uses by physiotherapy professionals	N/A	Recommendations for appropriate social media uses
Physiotherapy in social media… two sides of the same coin^36^	Samantha Schock	View Points	USA	2021	To express an opinion following the publication of ‘Making the Connection: A Guide to Social Media, Blogging, and Other Online Tools’ in APTA Magazine	N/A	Recommendations for appropriate social media uses
Physiotherapy in social media… two sides of the same coin^43^	Youness Azemmour	Editorial	Morocco	2021	To express an opinion about physiotherapy on social media	N/A	Development of ethical reflections on the topic
Physical Therapy 2.0: Leveraging Social Media to Engage Patients in Rehabilitation and Health Promotion^44^	Emily Knight, Robert J. Werstine, Diane M. Rasmussen-Pennington, Deborah Fitzsimmons, Robert J. Petrella	Conceptual article	Canada	2014	Presents author’s perspective that physical therapists may be able to leverage the power and utility of social media to enhance care delivery and treatment outcomes.	N/A	Advocacy and promotion of social media opportunities in physiotherapy
Analysis of Cyberincivility in Posts by Health Professions Students: Descriptive Twitter Data Mining Study^37^	Jennie C De Gagne; Eunji Cho; Sandra S Yamane; Haesu Jin; Jeehae D Nam; Dukyoo Jung	Quantitative study	United States	2021	To describe the characteristics and instances of cyberincivility posted on Twitter by users self-identifying as health professionals.	Descriptive cross-sectional data mining method of Twitter	Exploration of current social media practices
					To analyze the prevalence of tweets perceived as inappropriate or potentially reprehensible, while describing patterns and differences in cases of cyberincivility posted by these users.		
Ethical Considerations in Using Facebook for Health Care Support: A Case Study Using Concussion Management^38^	Osman Hassan Ahmed; Stephen John Sullivan; Anthony G. Schneiders; Lynley Anderson; Chris Paton; Paul R McCrory	Case study	New-Zeland	2013	To provide a non-exhaustive exploration of key ethical considerations in delivering a concussion management intervention through a Facebook group, and to discuss potential solutions to mitigate these issues	N/A	Recommendations for appropriate social media uses
Using social media to connect, facilitate communication, and practice knowledge translation^58^	Charlotte Wåhlin	Editorial	Sweden	2018	Share some thoughts on social media	N/A	Advocacy and promotion of social media opportunities in physiotherapy
News From NEXT: Oxford Debaters Argue: Is Social Media Hazardous?^34^	APTA Staff	Resume from NEXT: Oxford Debaters Argue	USA	2019	Resume from the outcome of the 12th annual Oxford Debate, during APTA’s NEXT Conference and Exposition in Chicago	N/A	Development of ethical reflections on the topic
New Technology: Keeping It Ethical, Keeping It Legal^49^	Michele Wojciechowsi	Article d’opinion	USA	2019	To propose possible responses to the ethical issues raised by the use of new technologies in physiotherapy	N/A	Recommendations for appropriate social media uses
Use of social media among Italian physiotherapists: a new opportunity for the profession or an unfavourable trend toward guruism?^51^	Stefano Vercelli	Letter to editor	Italy	2016	Propose theoretical frameworks to analyze the behaviour of users and the post contents on Italian pages dedicated to physiotherapy	N/A	Development of ethical reflections on the topic
Physiotherapy past, present and future. Embracing the digital age: physiotherapy and social media^53^	Hebron C	Qualitative study	UK	2018	Highlight the responsibility of physiotherapy educators to prepare students to critically evaluate evidence and assess the credibility of a source and value the opinions of all, particularly those who challenge us to think in different ways	Phenomenological study	Exploration of current social media practices
Top tips for social media use in sports and exercise medicine: doing the right thing in the digital age^39^	Osman Hassan Ahmed, Richard Weiler, Anthony G Schneiders, Paul McCrory, S John Sullivan	Editorial	UK	2015	Provide guidelines for using social media in sports and exercise medicine	N/A	Recommendations for appropriate social media uses
Student Focus^54^	APTA Casey Schlottman, Leada Malek, Marvin Jacob	Interview	USA	2022	Discuss student uses of social media.	N/A	Exploration of current social media practices
Use of Social Web and Perceptions of Professional Behaviours Regarding Social Web Postings among Physiotherapy Students^23^	K. Jothi prasanna Priyanka B	Quantitative study	India	2021	To survey students regarding their use of social media, social media postings, their judgments about the behaviours of others using social media and their own rating of privacy concerns	Cross-sectional exploratory study	Exploration of current social media practices
Therapists’ Perceptions of Social Media and Video Game Technologies in Upper Limb Rehabilitation^40^	Sandy K Tatla; Navid Shirzad; Keith R Lohse; Naznin Virji-Babul; Alison M Hoens; Liisa Holsti; Linda C Li; Kimberly J Miller; Melanie Y Lam; HF Machiel Van der Loos	Qualitative study	Canada	2015	To explore therapists’ perceptions of how young people and adults with hemiplegia use gaming and social media technologies in daily life and in rehabilitation, and to identify barriers to using these technologies in rehabilitation.	Exploratory focus group study	Exploration of current social media practices
Social media for physiotherapy clinics: considerations in creating a Facebook page^57^	Osman H Ahmed; Leica S Claydon; Daniel C Ribeiro; Ashokan Arumugam; Chris Higgs; G David Baxter	Opinion article	New-Zeland	2013	To provide an outline of the considerations involved in planning, implementing and maintaining a physiotherapy clinic Facebook page	N/A	Advocacy and promotion of social media opportunities in physiotherapy
Manage your online reputation^55^	Sandra Conrad	Opinion article	USA	2015	To help manage your online reputation, making social media work.	N/A	Advocacy and promotion of social media opportunities in physiotherapy
Physical Therapist Student Use of Social Media and Perceptions of Professional Behaviours Regarding Social Media Postings^25^	Renee Mabey; Peggy Mohr; Debbie Ingram; Thomas Mohr; and Laura Lee (Dolly) Swisher	Quantitative study	USA	2019	To survey students enrolled in professional physical therapy programs regarding their use of social media, the types of materials they and others posted, their judgments about behaviours of others using social media, and their rating of privacy concerns given different clinical scenarios	Cross-sectional exploratory study	Exploration of current social media practices
Plugging into social media^45^	Jonathan Simkins	Opinion article	USA	2017	Present how to take advantage of using social media in PT	N/A	Advocacy and promotion of social media opportunities in physiotherapy
LinkedIn or out? Can social media platforms be useful to POGP members?^35^	A. Savage	Opinion article and conference discussion summary	UK	2016	To discuss and response to Graham Aikin’s talk about social media at the 2015 Pelvic, Obstetric, Gynaecological Physiotherapy Annual Conference	N/A	Advocacy and promotion of social media opportunities in physiotherapy
Ethical guidelines and the use of social media and text messaging in health care: a review of literature^33^	Rachel Basevi, Duncan Reid, Rosemary Godbold	Litterature review	New-Zeland	2014	To undertake a review of the literature investigating the application and use of social media, guideline developments, and analyzing the key ethical issues identified by this review.	Litterature review	Recommendations for appropriate social media uses
The Perils of Posting a PT get exercised, with problematic results^56^	Nancy R. Kirsch	Cases study	USA	2019	Analyze a case study on an online situation and to consider issues raised by the scenario.	N/A	Development of ethical reflections on the topic
Making the Connection: A Guide to Social Media, Blogging, and other Online Tools^49^	Michele Wojciechowsi	Guideline	USA	2021	Give information and tips for beginners	N/A	Recommendations for appropriate social media uses
Keep posting and following social media profiles about physical therapy, but be aware! A cross-sectional study of social media posts on Instagram and Twitter^52^	Bruna Wageck; Iris S. Noal; Brenda D. Guterres; Samantha L. Adami; Daiane Bordin; Mauricio Fanfa; Guilherme S. Nunes	Quantitative study	Brasil	2023	To systematically examine social media posts that disclose information related to physiotherapy interventions and to assess: (i) whether the posts mention information sources to support their content; (ii) the presence of elements that may suggest a conflict of interest (COI); (iii) whether the information is presented in a way that facilitates knowledge acquisition; (iv) the scope of the information shared; and (v) the use and quality of the scientific references cited.	Cross sectional study	Exploration of current social media practices
Development and validation of self-assessment instrument to measure the digital professionalism of healthcare professionals using social media^48^	Shazia Imran, Rahila Yasmeen and Memoona Mansoor	Mixed-method study	Pakistan	2024	To identify the key domains and elements that enable an adequate assessment of digital professionalism among health professionals using social media	Instrument development based on a review of clinical practice guidelines, followed by content validation through the Delphi method	Development of an instrument to assess digital professionalism
					To validate a self-assessment tool designed to measure digital professionalism of health professionals on social media		
Using Twitter (X) to Mobilize Knowledge for First Contact Physiotherapists: Qualitative Study^41^	Laura Campbell; Jonathan Quicke; Kay Stevenson, Grad Dip Phys, MPhil; Zoe Paskins; Krysia Dziedzic; Laura Swaithes	Qualitative study	UK	2024	To explore how Twitter is used, and can be used, to mobilize knowledge in order to inform clinical practice among physiotherapy professionals (PTPs). The mindlines model is employed as a lens to explore and understand the social processes associated with how PTPs use different types of knowledge from Twitter to inform their mindlines.	Semi-structured interviews	Exploration of current social media practices
#Boundaries: When Is the Line Being Crossed?^42^	Debra Gorman-Badar and Rebecca Edgeworth Ditwiler	Case study	USA	2023	Not explicited	Case description and analysis	Development of ethical reflections on the topic
